# Supplementary material for: A Transmembrane Histidine Kinase Functions as a pH Sensor
Source: Biomolecules. 2020 Aug 14;10(8):1183. doi: 10.3390/biom10081183 (PMC7465017; doi:10.3390/biom10081183)
Supplement: Supplementary file 1 [file biomolecules-10-01183-s001.pdf]

Supplementary Materials:

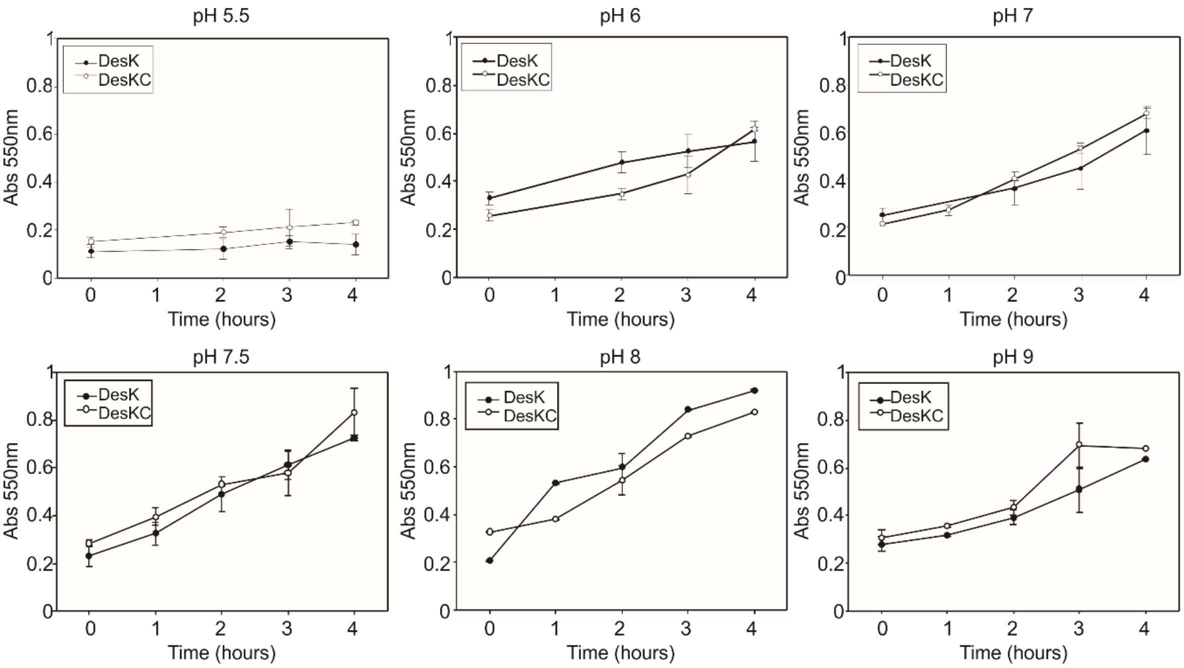

Figure S1. Growth curve for the indicated strains at 25 °C. Cells were cultivated for four hours in minimal medium (SPI) adjusted at the different pHs. The density of the culture was determined at each hour using the OD550.

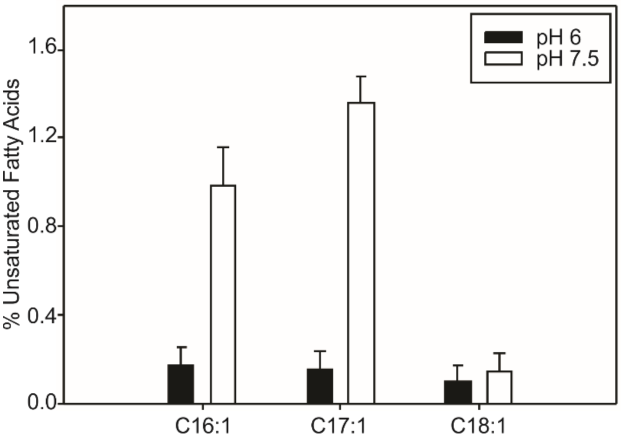

Figure S2. *Bacillus* cells expressing MS-DesK were grown at a constant temperature (25 °C) but different pHs. The UFA content was determined by gas chromatography

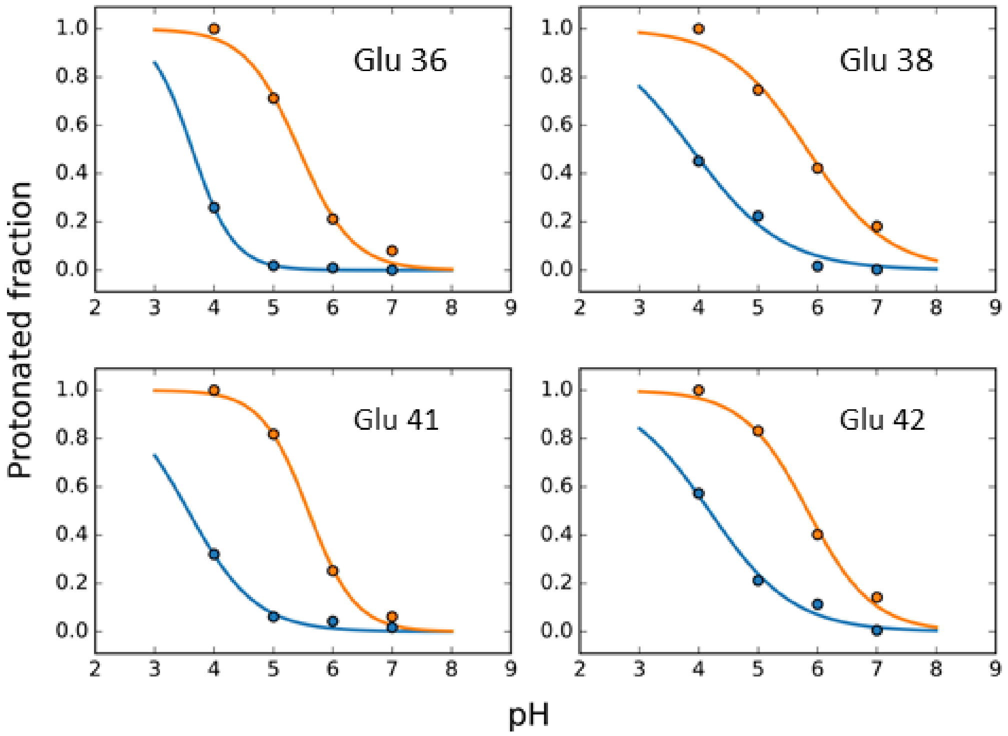

Figure S3. Titration curves of glutamate amino acids at pHs ranging from 4 to 7. The blue curve corresponds to the linker in bulk solution, and the orange curve corresponds to the linker close to the surface of the membrane.

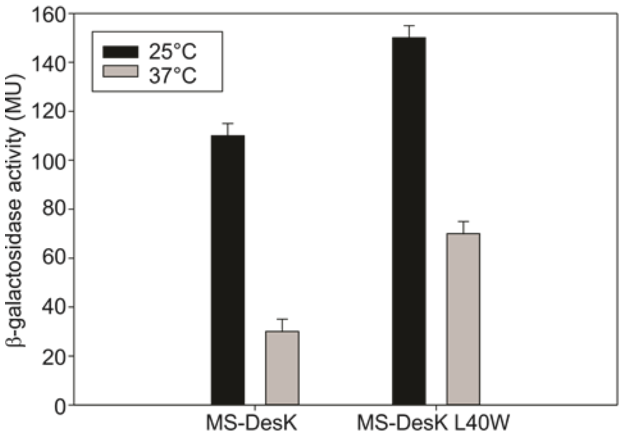

Figure S4. *Bacillus* strains CM21 expressing MS-DesK and MS-DesK L40W were grown at 25 °C and 37 °C. Activity of the reporter gene was measured as in Fig 1. Error bars include the standard deviation from at least five independent experiments**.**

Supplementary Material and Methods:

Mutagenesis.

Site-directed mutagenesis was performed to introduce mutations in MS-DesK using plasmid pHPKS-TM1/5DesKC. For the PCR was used Q5 polymerase high fidelity from New England Biolabs (NEB) and the primers were from Tecnolab, Argentina. The resulting plasmids were used to transform CM21 *B. subtilis* [13]. This strain is a DesK mutant and contains a transcriptional fusion between the reporter gene β-galactosidase and

the promoter of the desaturase (gene upregulated by DesK–DesR at low temperature), which allows for monitoring kinase activity. Mutations were confirmed by DNA sequence analysis by MACROGENE. Strains, full sequences and detailed construction methods are available upon request.

Fatty acids analysis.

Bacillus cells were grown at the different pHs in Spizizen medium (already described). Lipids were extracted by the Bligh and Dyer technique and fatty acids converted to their methyl esters with sodium methoxide, purchased from Sigma Aldrich [1]. The methyl esters were run in a Perkin-Elmer Turbo mass gas chromatographer-mass spectrometer, equipped with a PEG column, and PerkinElmer software. Each fatty acid was identified by comparing its mass spectrum with those obtained from methyl esters of fatty acid standards (Sigma Aldrich, Inc.).

Molecular dynamics.

The initial model of the linker was created as an alpha helix using Modeller9.15 [2] based on its sequence. The lipid bilayer was created using charmm-gui [3], with a composition of 75% phosphatidylglycerol and 25% phosphatidylethanolamine. One system was built in which the protein was immersed in water with 0.15M KCl and another one in which the linker was placed on the surface of the membrane surrounded by water with the same concentration of KCl. Molecular dynamics Simulations were performed using the PMEMD module of the AMBER18 package with the FF14SB force field for the protein [4], Lipid14 for the lipids [5] and explicit TIP3P for water [6]. The simulation protocol consisted of an energy minimization stage, a thermalization stage in which the system was taken from 0K to 300K at constant pressure and constant surface tension during 1ns, and finally a simulation at 300K and constant pressure and surface tension during 100ns. The final frame of the production run was used as a starting point for constant pH molecular dynamics during 100ns at pHs from 4 to 7. In this type of simulation [7], the titratable residues change their protonation state according to its electrostatic environment and the pH at which the simulation is set [8]. The pKa was calculated by adjusting the protonated fraction at different pHs to the Henderson–Hasselbach equation using the SciPy module of Python.

Reference

1. Bligh, E.G. and Dyer, W.J. Canadian Journal of Biochemistry and Physiology. *Can. J. Biochem. Physiol.* **1959**, *37*.
2. Webb, B.; Sali, A. Protein Structure Modeling with MODELLER. *Adv. Struct. Saf. Stud.* **2014**, *1137*, 1–15.
3. Jo, S.; Kim, T.; Iyer, V.G.; Im, W. CHARMM-GUI: A web-based graphical user interface for CHARMM. *J. Comput. Chem.* **2008**, *29*, 1859–1865, doi:10.1002/jcc.20945.
4. Maier, J.A.; Martinez, C.; Kasavajhala, K.; Wickstrom, L.; Hauser, K.; Simmerling, C.L. ff14SB: Improving the Accuracy of Protein Side Chain and Backbone Parameters from ff99SB. *J. Chem. Theory Comput.* **2015**, *11*, 3696–3713, doi:10.1021/acs.jctc.5b00255.
5. Dickson, C.J.; Madej, B.D.; Skjevik, Åge, A.; Betz, R.M.; Teigen, K.; Gould, I.R.; Walker, R.C. Lipid14: The Amber Lipid Force Field. *J. Chem. Theory Comput.* **2014**, *10*, 865–879, doi:10.1021/ct4010307.
6. Price, D.J.; Brooks, C.L. A modified TIP3P water potential for simulation with Ewald summation. *J. Chem. Phys.* **2004**, *121*, 10096, doi:10.1063/1.1808117.

7. Drusin, S.I.; Rasia, R.M.; Moreno, D. Study of the role of Mg2+ in dsRNA processing mechanism by bacterial RNase III through QM/MM simulations. *JBIC J. Boil. Inorg. Chem.* **2019**, *25*, 89–98, doi:10.1007/s00775-019-01741-7.
8. Swails, J.M.; York, D.M.; Roitberg, A.E. Constant pH Replica Exchange Molecular Dynamics in Explicit Solvent Using Discrete Protonation States: Implementation, Testing, and Validation. *J. Chem. Theory Comput.* **2014**, *10*, 1341–1352, doi:10.1021/ct401042b.
